# Leveraging OCR and HTR cloud services towards data mobilisation of historical plant names

**DOI:** 10.1007/s42803-024-00091-4

**Published:** 2024-11-28

**Authors:** Jawad Sadek, Andreas Vlachidis, Victoria Pickering, Marco Humbel, Daniele Metilli, Mark Carine, Julianne Nyhan

**Affiliations:** 1 Department of Information Studies, University College London, London, UK; 2 National History Museum, London, UK; 3 Technische Universität Darmstadt, Darmstadt, Germany

**Keywords:** OCR, Handwritten text recognition, Historical documents, Marginalia annotation, Botanical specimen, Herbarium

## Abstract

We present our solution to the problem of how to mobilise (that is, extract and enrich) digital data from the analogue, printed book version Sir Hans Sloane’s copy of John Ray’s Historia Plantarum, to create the first searchable facility of its kind to the plants contained in the Sloane Herbarium, housed in the National History Museum UK. The data mobilisation workflow presented here enables the automatic detection of printed and handwritten marginalia text and annotations in Sir Hans Sloane” personal copy of John Ray’s Historia Plantarum. The rationale of adopting AWS Amazon’s Textract service and the development of a specialised information extraction workflow for mobilising printed text and handwritten annotations is discussed. Testing of our workflow demonstrates the need for human-checking of outputs to ensure the accuracy of a large set of structured data comprising 7600 plant names and 4540 handwritten marginalia annotation. The links we have created serve as the first digital index to Sloan’s Herbarium, a unique development in the longer analogue and digital format-history of these resources.

## Introduction

Sir Hans Sloane (1660–1753) is known for being a physician, naturalist and collector, as well as an individual who financially profited from the transatlantic slave trade, and who established a vast and varied collection of objects and things during his long life. As the foundation collection of the British Museum, Sloane’s collection is considered part of the founding collection of the UK cultural heritage sector. Consequently, and as part of the AHRC-funded *Towards a National Collection* (TaNC) programme, the Sloane Lab project, launched in 2022, is facilitating digital access to the historical and present-day information that describes the objects collected by Sloane. As this paper will discuss, the Sloane Lab is enabling digital access to the Natural History Museum’s (NHM) Historical Botanical Collection in a way that has not been possible before (Sloane Lab, 2024).

Over the course of several decades, Sloane amassed hundreds of thousands of natural history objects such as shells, fish, coins and manuscripts. Collection management was of crucial importance to Sloane, especially for the preservation and future use of the collection he was amassing (Caygill, 2012). The British Museum (BM) opened to the public in 1759, using Sloane’s collection as part of its founding collection. Over a century later, the NHM was opened in South Kensington, a site to house and display the BM’s increasing collection of natural history objects. The NHM’s Historical Botanical Collection includes the Sloane Herbarium which is the largest surviving part of Sloane’s natural history collections. The Sloane Herbarium, which is located in the NHM’s Darwin Centre in a specially built room, consists of around 121,000 botanical specimens which have been dried, pressed, and mounted onto sheets of paper and bound into 337 *Horti Sicci* (“dry gardens”). These are numbered, with the first seven comprising over 1200 specimens, largely gathered by Sloane while he was in Jamaica between 1687–89 as physician to the governor. In total, the plant specimens in the herbarium were collected and contributed by over 300 named individuals. While Sloane lived and worked in London, he engaged in a vast global network of correspondence that enabled him to accumulate a botanical collection of this scale. In some instances, Sloane acquired the entire collections of others and in others, he benefited from trading networks and colonial expansion (Carine, 2020).

The Sloane Herbarium is ‘pre-Linnean’ which means that the volumes within it were originally formed, arranged and documented before the binomial system of naming that is still in use today was adopted following the publication of the first edition of Carl Linnaeus’ *Species Plantarum* in 1753 (Von Linne & Salvius, 1753) The Sloane Herbarium is one of the largest surviving pre-Linnean collections and is significant for a range of research including taxonomy, understanding environmental change and conservation, investigating plant use, ecology as history of science, art and literature, teaching, and outreach. Such a collection is constantly attracting new users (Carine et al., 2018).

Ray’s *Historia Plantarum* is a three-volume work in Latin. Volumes I and II were published in 1686 and 1688 and contain some 1,000 pages each. Volume III is a supplementary volume of a similar size, published in 1704. It is a work that aimed to create a world-wide encyclopaedia of the flora then known to European Natural Historians, listing and describing the species identified at that time. In the *Historia Plantarum*, Ray classified approximately 18,000 plant species with details about their structures, anatomy, and botanical differences. Common names are often provided and descriptions typically also include details related to habitats, times of flowering, whether annual or perennial as well as medicinal properties (Carine, 2020).

Sloane and his assistants used Sloane’s personal copy of Ray’s *Historia Plantarum* to classify and catalogue the plant specimens found throughout the 337 *Horti Sicci* in his herbarium. This copy, which is also housed within the NHM’s Historical Botanical Collection, contains a plethora of handwritten annotations and markings, and it is these markings, along with the list names of plants contained in that work, that the Sloane Lab has focused its ‘data mobilisation’ (or efforts to extract digital data from and analogue source and further enrich it so as to make it machine readable) efforts on.

**Fig. 1:**
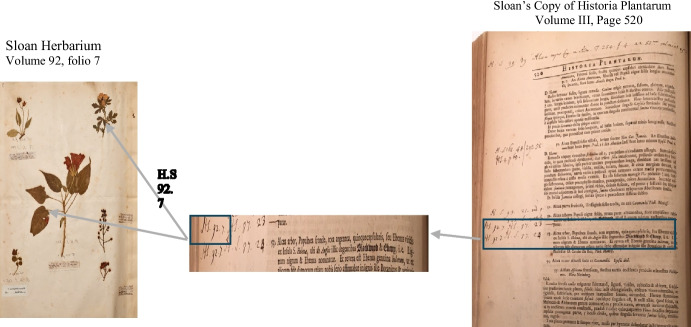
Handwritten annotations in Historia Plantarum reference plant names to specimens in Sloane herbarium

In the side margins of the folios of these volumes, we find handwritten notations that are next to, or, in very close proximity to a plant name. These annotations take the form of ‘H.S. *[Hortus siccus* number] [folio number]’ as illustrated in Fig. 1. The combination of *Hortus siccus* number and folio number acts as an indexing reference to the physical location of a specimen within the herbarium. Folios may contain more than a single specimen and occasionally an additional number is included to the reference pattern following the folio number that specifies the exact location of a specimen on a folio or page, typically where the specimens on a page are themselves numbered. At the top and bottom margins of the pages, also referred to as *headers* and *footers,* we find additional handwritten annotations containing plant names and indexing references to the herbarium following the same volume and folio format found within the side margins. Here, the plant species names are those not already included within Ray’s list (Ray, 1688). The marginal annotations together with the headers and footers provide the most comprehensive (albeit still partial) index to the Sloane Herbarium. As Delbourgo and Müller-Wille argue, such a document ‘captures the interplay of divergent material regimes of scientific paperwork: the ostensibly fixed printed list and the recurrent manual inscription required to update it (Delbourgo & Müller-Wille, 2012).

Until this work was undertaken, to search for specimens of a particular species in the Sloane Herbarium, a user had to be physically located in the NHM and able to access Sloane’s copy of *Historia Plantarum*. This not only presents physical challenges, but a user must also hold relevant botanical and historical expertise and knowledge to be able to navigate different taxonomic systems. Notably, a user must be able to make connections (or spot divergences), between the polynomial plant name descriptions found in the eighteenth century and Ray’s *Historia Plantarum* with current binomial nomenclature.

Digitisation is an increasingly important focus for herbaria. Digitising collections and making them globally and freely available, has the potential to accelerate their long-established use for generating primary biodiversity data while also allowing their data to be unlocked for novel uses, scientific, historical and cultural (Davis, 2023).

The Sloane Herbarium has, at times, been overlooked in terms of its research potential and significance, in part because it is pre-Linnaean and difficult to use and navigate (MacGregor & McAlpine, 1994). The extraction of information about plant names and the association of corresponding physical location codes (the volume and folio numbers) makes the Sloane Herbarium digitally searchable for the very first time. Doing so will have a range of benefits. They include: helping to unlock the potential of the collection to address a range of questions such as changes in the distributions of plants, including their extinction, over the last three hundred years, a period of unprecedented environmental change; enriching our understanding of human kind’s relationships with plants, how we have domesticated them and used them as sources of medicines; understanding the complex relationship between natural history, trade and colonialism, including the trade in enslaved people; potentially opening new uses for this unique collection.

To lay the data foundation for such questions to be pursued extracted plant names are represented through the Sloane Lab Data Model (Metilli et al. forthcoming) and then integrated into the Sloane Lab Knowledge Base (KB), a web application and knowledge graph that allows users to query, navigate and visualise the datasets aggregated by the Sloane Lab project, including items now housed not only at the Natural History Museum but also the British Museum and British Library. Through the KB, users can explore each volume of the Historia Plantarum and, through the transcription of the handwritten annotations, understand how it relates to the Sloane Herbarium.

Thus, we aim to mobilise Sloane’s copy of Ray’s *Historia Plantarum* to enable the Sloane Herbarium to be digitally searchable for the first time. In this study, we focus on processing the digitised images of volume I and volume II which contain the majority of plant name location codes to the herbarium. The specific objective of this part of the workflow is to extract three orders of information: (i) the plant names which appear in the main printed body of the text, (ii) the handwritten annotations that are found in headers, footers and side margins, and finally (iii) the relationship between plant names and their corresponding specimen annotations. Existing off- the-shelf software cannot comprehensively or accurately automate this process of data mobilisation for several reasons. Firstly, the handwritten annotations and numbers which indicate the specimen locations in Sloane’s Herbarium appear in different hands. It is crucial that these numbers are recognized with the highest degree of accuracy to ensure that specimens can be correctly located in the herbarium using the digital resource generated. Secondly, associating plant names with their respective specimens poses unique challenges that cannot be captured by generalised software, as discussed below. Consequently, a customized solution to mobilise data was required. Our approach to this task involved multiple stages, incorporating automatic text recognition services and rule-based algorithms.

The remainder of the paper is organised as follows: Section 2 explores the wider research context of digital botany projects, highlighting challenges inherent in digitising historical documents using Optical Character Recognition (OCR) and Handwritten Text Recognition (HTR) techniques. Section 3 outlines the methods we adopted to solve our data mobilisation requirements. Section 4 evaluates the performance of those methods and in Section 5 synthesises and analyses our findings and provide recommendations for further progress.

## Research context

DH and Digital Cultural Heritage (DCH) projects have been increasingly integrating computational approaches to analyse and preserve diverse cultural and historical content. These techniques enable the development of digital tools and platforms for enhancing access, interpretation, and engagement with cultural heritage materials. These computational approaches are valuable in the botanical domain, where vast amounts of data about plant species and historical botanical collections can be digitised, analysed, and made accessible to a broader audience. In the subsequent sections, we provide an overview of botanical projects, the computational techniques they employ, the challenges they face and the current advancements in this field.

### Botanical projects and technology

There are around 3000 herbaria worldwide that collectively comprise an estimated 400 million specimens (Thiers, 2022). Those specimens can be considered a vast, globally distributed infrastructure for investigating plant diversity through time. Digitising and connecting those collections to facilitate access and research has been, and remains, a major focus for herbaria worldwide, with initiatives at the institutional (Barkworth & Murrell, 2012), national (About - UK Collections, n.d.; About iDIGBio | iDigBio, n.d.) and international levels (DiSSCo, 2024; Smith & Figueiredo, 2014). Alongside specimen digitisation, initiatives to digitise relevant biodiversity literature (Botanicus.org, n.d.; Gwinn & Rinaldo, 2009) and to link data across institutions and data types (Hardisty et al., 2022) are advancing at pace.

Robust digitisation workflows and agreed data standards (Carine, 2020) have been integral to the digitisation and integration of natural history collections at scale. Herbarium specimens are typically two dimensional and pressed on standard sized sheets to facilitate high-throughput imaging. They will typically be labelled with a standard set of data that can be captured from the specimen and which includes the Linnean binomial name for the plant (often with the author of that name); the name(s) of the collectors and sometimes a collection number; the date on which a specimen was collected and the site at which it was collected. The use of machine-readable barcodes attached to herbarium sheets facilitates the linking of those data and images.

Projects heavily rely on computational methods for tasks ranging from digitisation and data extraction to analysis and visualisation. Some primarily rely on digitisation efforts to make physical archives more readily available. Others incorporate advanced algorithms and machine learning techniques to extract hidden patterns, relationships, and insights from vast amounts of data. For instance, the US Virtual Herbarium heavily relies on computer vision and pattern recognition algorithms to classify and index large collections of herbaria sheets automatically. On the other hand, Owen et al. (2020) tested different tools and services to implement their digitisation and transcription pipelines. Their test results showed that employing Tesseract 4.0.0 for image segmentation task and Google Cloud Vision for HTR processing led to promising text recognition accuracy. They also evaluated Stanford, a pre-trained machine learning classifier, on a Named Entity Recognition (NER) task. Stanford NER achieved an F1-score of 0.71 in extracting People and Locations categories from segmented images. Cheng et al. (2023) trained R-CNN and Faster R-CNN networks to predict coordinates of handwritten marginalia. They also developed an algorithm for segmenting handwritten text to individual words where each segmented word fed into a pre-trained model. Lehenmeier et al. (2020) used the Transkribus platform to create labelled data for training and evaluation. The ground truth data was then used to train text recognition models with Calamari-Ocr (n.d.). The authors reported that their system achieved an accuracy of 87% for layout and table recognition and 82% accuracy for handwritten text recognition. An excellent result, but nevertheless insufficient as an entirely automated workflow for use in research contexts, where accuracy levels in excess of 99.5% (Deutsche Forschungsgemeinschaft, 2016, p. 37) are required.

### OCR & HTR

OCR engines are based on pattern recognition, machine learning and image processing algorithms. They typically learn from large datasets of printed text in various fonts and sizes. To achieve accurate results, OCR requires preprocessing steps, such as image cleaning, fixing alignment issues and removing unwanted elements. In the context of herbarium collections, OCR helps digitise printed information from specimen labels, including details about plant species, collection information, and measurement references.

HTR engines use deep learning techniques to model the sequence of strokes and characters in handwritten text. They need large amounts of annotated handwritten text to train models. Like OCR, HTR engines require image preparation, with additional focus on understanding pen strokes, orientation, and character segmentation. HTR accuracy is generally lower than OCR due to handwriting variations. Yet HTR engines are particularly valuable for processing older herbarium collections where labels are often handwritten.

The combination of OCR and HTR technologies can make botanical specimen information more digitally accessible. The digitisation of these labels allows researchers to conduct research on biodiversity trends, historical botanical data, and environmental changes over time, such as discussed above. Additionally, measurement references, such as rulers and size indicators, located on herbarium specimens play a crucial role in analysing plant samples; OCR can be used to recognize numeric values and units of measurement.

Recent advancements in HTR and OCR have been primarily driven by deep learning architectures and transformer-based models (Ströbel et al., 2023). The integration of attention mechanisms and transformer networks, as demonstrated by Li et al. (2023) in their work on TrOCR, has improved accuracy in both printed and handwritten text recognition. A notable breakthrough came with the development of DocFormerV2 (Appalaraju et al., 2024), a multi-modal transformer architecture which combines visual and textual features in a unified architecture for information extraction and form recognition achieving state-of-the-art results on multiple benchmark datasets.

### Cloud-based OCR & HTR platforms

Many cloud services provide OCR and HTR solutions that can recognise text in multiple languages. These services have become increasingly popular in recent years due to their ease of use, scalability, and cost-effectiveness. Some platforms offer cloud-based OCR services as part of their machine learning tools including Amazon Textract (Amazon Textract, n.d.) provided by Amazon Web Services (AWS), Azure Cognitive. (Microsoft Azure, n.d.) provided by Microsoft Azure and Google Cloud Vision (Vision AI, n.d.) provided by Google Cloud Platform (GCP). Other platforms are designed particularly to deal with text recognition for example Transkribus (Transkribus, n.d.) and Abbyy Cloud (Abbyy, n.d.).

Developers can typically access these services via an API or web interface and pay for their usage based on the volume of OCR processing they require. Cloud-based OCR services offer several benefits over on-premises OCR solutions. One of the main advantages of using OCR cloud services is the ability to scale processing resources up or down as required without the need for dedicated hardware or IT resources. This can be particularly useful for research projects such as the Sloane Lab, which operate within constrained timeframes as it provides development agility and enables them to easily adapt to changes in demand. Additionally, cloud services can also offer a range of features, such as language recognition, support for multiple languages including early modern English and Latin (which are the focus of our research), and the ability to train custom models for specific use cases. This capability can be particularly useful for building a machine learning model trained on transcribed handwritten scripts.

Heritage institutions can benefit significantly from Cloud-based OCR to unlock the potential of their collections, by reducing operational costs associated with equipment maintenance, software licences, and personnel training. The scalability and flexibility of cloud OCR also allows institutions to process large volumes of documents efficiently. However, it is important to consider the cost and security implications of using a cloud service for OCR. Documents and data are processed by the vendor while data is in transit, so it is important to ensure the service provider has appropriate security measures in place to protect the data, and that they comply with relevant data privacy regulations, such as GDPR or CCPA. Additionally, cloud-based OCR relies on the service provider to remain operational so if there are technical issues on the vendor side, projects may lose control over the service.

Our approach leverages the results generated by AWS Textract, ensuring the precise capture of relevant information. AWS Textract offers pre-trained ML models that extract printed and handwritten text from various document types, eliminating the need for manual setup or extensive data training. It goes beyond basic OCR by identifying and extracting data from forms and tables. Since our project is already integrated with AWS cloud services, by using Textract we benefit from its native integration with other services like S3, Amazon comprehend, and Augmented AI streamlining data workflows. Moreover, Textract returns the bounding box coordinates for each word level in documents which is a key feature for the aims of this study which is further discussed in Section 3.2.

### Computational challenges

There are several challenges when it comes to recognizing and extracting text from historical resources, particularly when dealing with older or degraded documents. Older documents may have undergone physical deterioration over time, causing torn pages, faded ink, or stains that can make it difficult for OCR systems to accurately interpret text. The typefaces or handwriting styles used in historical documents can also vary significantly from modern standards. Additionally, these documents may contain handwritten annotations or marginalia which can further complicate the text extraction process.

Many of these challenges are also present when extracting texts from *Historia Plantarum*. The page quality for instance poses issues, as some pages are faded or degraded. Additionally, the book contains stained text and other imperfections such as tears or discolorations. These issues can make it difficult for an OCR system to accurately recognise characters. Furthermore, many pages have non-standard text layouts, such as multiple columns, decorative text elements, and unusual margins which can be challenging for text recognition. The OCR must accurately differentiate between actual text and other elements such as borders. Additionally, parts of the adjacent pages may appear on the scanned images which requires preservation of the original layout to maintain the readability and integrity of the document.

Text in *Historia Plantarum* presents challenges due to its use of irregular fonts and language variations. The text often features typography that has evolved significantly over time, including Gothic fonts, ornate handwritten scripts or abbreviations that refer to specific authors. Software can struggle to decipher scripts with unique features. Additionally, *Historia Plantarum* is written in Latin, a language that is no longer in use which introduces further complexities. Inconsistencies in spelling and grammar within the text add to the difficulty, making it harder for modern OCR to accurately interpret the content, even when incorporating specialised dictionaries into routines.

OCR and HTR systems rely heavily on training data to recognize printed text. However, when it comes to Latin and Early Modern English, there is a scarcity of digitised and annotated texts from these eras. These periods feature distinct linguistic characteristics, including unique vocabulary, spelling variations and archaic grammar structures. This scarcity of training data can lead to frequent errors. To overcome these challenges, OCR systems require careful consideration and specialised techniques that can address these challenges. In many cases it is essential to have a post processing stage and manual interventions by human experts to ensure accurate results and improve accuracy (Cheng et al., 2023; Lehenmeier et al., 2020). Consequently, to capture data from the *Historia Plantarum,* it was essential to employ a customised pipeline designed specifically to meet its unique requirements.

## Methodology

In this section, we present a comprehensive overview of the methodology adopted in this study to mobilise data from Sloane’s copy of John Ray’s *Historia Plantarum*. We explain this study’s methodological basis and highlight the key considerations that guided our approach to extract the relevant information i.e. capturing plant names, handwritten annotations that appear in marginalia and the relationship between plant names and their corresponding specimens’ annotations.

### Proposed workflow

**Fig. 2:**
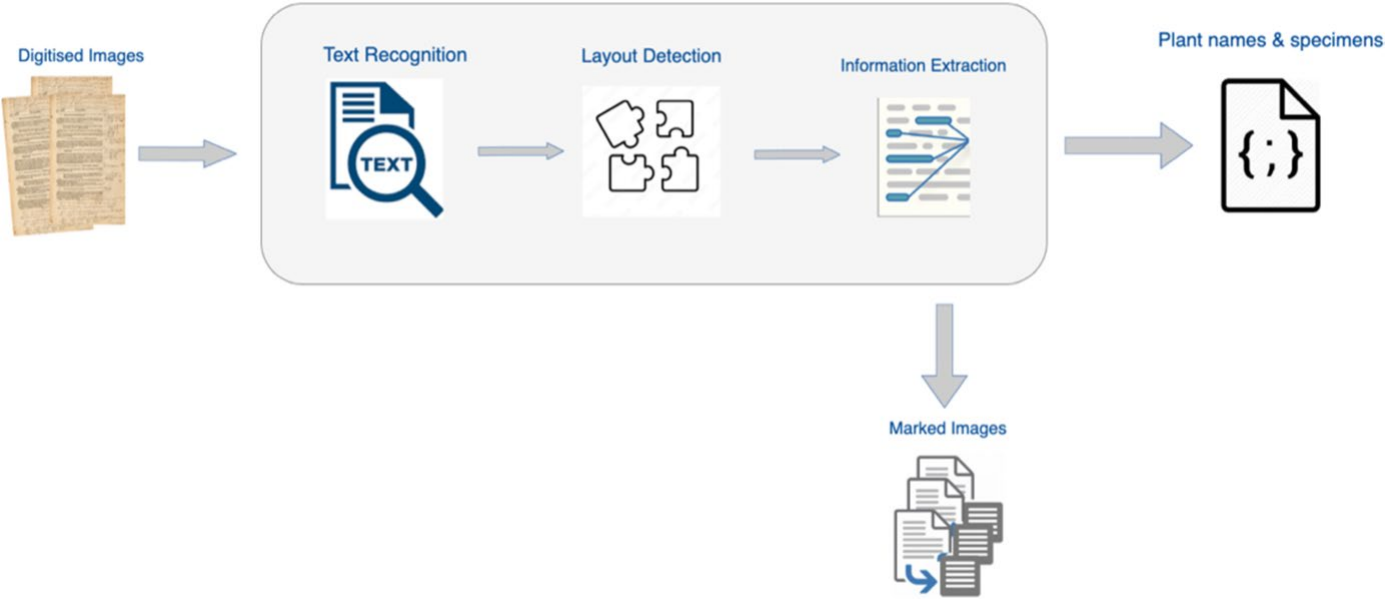
Data mobilisation workflow

We approached the data mobilisation task through the workflow presented in Fig. 2. The workflow was designed to leverage the output generated by Textract and align with the research objectives detailed in Section 1. The input is digitised images, where each image represents a single page of *Historia Plantarum*. The output is twofold: first, well-structured data that includes transcriptions of plant names and their associated handwritten references; second, a corpus of image segments of headers, footers, plant names and handwritten references. The processing pipeline in this workflow proceeds in three main stages: the first stage is **text recognition,** which includes the automatic transcription of the printed and handwritten text on each page using OCR and HTR techniques. The second stage is **page structure detection**, in which the customised structure and the layout of the document is identified, and the third stage is **segment and association detection,** where plant name entities are extracted and the relationship between these entities and their corresponding handwritten annotations are captured. It is important to note that Textract implicitly performs page layout detection prior to executing OCR/HTR. Our second stage involves customised analysis of Textract's output to recognise the relevant information for our study. Each stage is detailed in the following sections.

### Text recognition

In Section 2.2, we highlighted the availability of various OCR tools, each offering unique features and functionalities that suit the requirements of different research projects. Among these tools, Transkribus stands out as the preferred choice, at the time of writing, in the cultural heritage field (Nockels et al., 2022). Transkribus returns the bounding box coordinates for each line and text region (a rectangular frame that fully encompasses each piece of text identified) of the transcribed documents. However, it doesn’t offer bounding box coordinates at the world level. Obtaining word-level bounding box coordinates is essential for the data mobilisation task which must maintain a linked relationship between plant name and folio reference as found in the marginalia of the plantarum pages. The Amazon Textract output achieves this finer granularity and is accordingly adopted as the main tool of the Text recognition stage.

Amazon Textract is a machine learning service provided by AWS that has been trained on millions of documents so that it automatically extracts printed and handwritten text, and data from generic structured or unstructured documents (Amazon Textract, n.d.). Textract has two main operations: *“Detect document text”* which returns text detected in a document and *“Anaylze document”* in which it identifies and extracts data from forms and tables and finds relationships among detected text. The document structure and the relationships between printed and handwritten text in *Historia Plantarum* does not fit this model and the output of the *“Anaylze document*” operation does not meet the requirements defined for our task. Hence, we set custom rules to leverage the output generated by the *“Detect document text”* operation to accurately extract the image fragments representing the printed plant names and handwritten annotations and to also capture the relationships that connect these fragments.

When the *“Detect document text”* operation processes a document, the results are returned in an array of Block objects (PAGE, LINE and WORD). These objects represent lines of text or textual words that are detected and transcribed on a document page. Each object contains information about the detected text items, including the lines and words of the item, the relationships between the lines and words of the item, the page that the detected text appears on and the location of the lines and works of text on the document page.

**Fig. 3:**
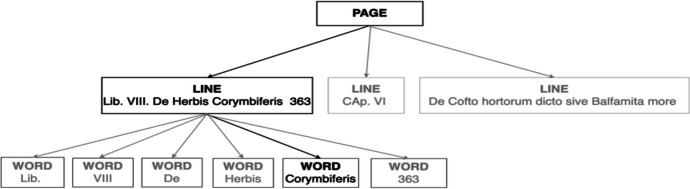
Lines from *Historia Plantarum* represented by Block objects

**Fig. 4:**
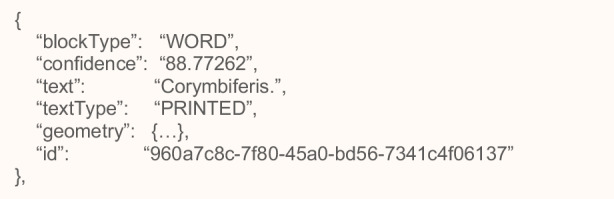
Textract output for the WORD block that represent the word “Corymbiferis”

Blocks are related to each other in a parent-to-child relationship. A ‘PAGE’ block is the parent for all ‘LINE’ block objects on a document page (Figs. 3 and 4). Because a ‘LINE’ block can have one or more words, the Relationships array for a ‘LINE’ block stores the IDs for child ‘WORD’ blocks that make up the line of text. Also, Textract determines if a piece of text was handwritten or printed, using the *‘Text Types’* field which is a valuable property in identifying the marginal borders of the processed page as explained in Section 3.4.

### Page structure detection

When using Textract, all extracted printed text, handwriting, and structured data are returned with bounding box coordinates. This allows us to identify the geometry points of each extracted entity so that we can make informed decisions about how each bounding box relates to the overall structure and the layout of each page.

During the algorithm development for page layout detection, a sample of pages representing different layout styles of *Historia Plantarum* were examined and analysed to identify distinct patterns and thus enable the recognition and extraction of relevant page segments. This process was iterated with new samples to enhance the algorithm performance until no further improvement was attainable. The algorithm processes each page in two sequential steps:

#### Determine the page group

Even and odd pages exhibit distinct characteristics regarding the placement of side margins, page numbers positions and header titles, necessitating separate processing for each. This step includes determining whether it is an odd or even numbered page in process. It involves creating customised rules to recognise the page number by detecting patterns and features that occur surrounding the page number location, such as page titles or the occurrence of specific initials. For instance, “Lib IV.“ “De Herbis” appear on odd-numbered pages only, while “HISTORIA PLATARUM” appears on even-numbered pages. There are variations in initials and titles depending on the *Historia Plantarum’s* Volumes and Sections. Additionally, inconsistencies in transcriptions should be considered by applying Regular Expressions patterns; for instance, the word “HISTORIA” might be transcribed as “H1STORIA”, “H IST OR1A” or “H I S T O R I A” etc.

#### Detect the structure of the page

The next step involves processing the image based on the page number group detected in the previous step. During this phase, the algorithm uses the geometry coordinates of the page provided by Textract to identify the positions of headers, footers, and side margins. For instance, by analysing the X-coordinates of all lines, the algorithm can determine where the side margins start. This process eliminates any text that may appear from adjacent pages and recognises the borders of the page, concluding its overall layout.

The output of the Page structure detection stage consists of page fragments of headers, footers, side margins, and the printed content of each page. Each fragment is structured data that contains the transcribed text for that fragment, along with the additional information provided by the “*Detect document text”* operation from Textract, as described in Section 3.2. Additionally, the corresponding image segment for each fragment are produced for further processing in the subsequent stage.

### Segments and association detection

This stage involves analysing the transcribed text and its associated geometry data to capture all paragraphs and plant names - handwritten annotation pairing in the extracted text.

**Fig. 5:**
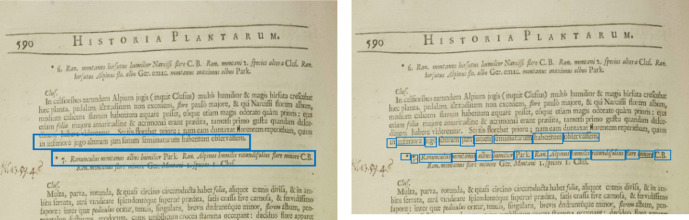
The bounding boxes of lines Vs bounding boxes of words. (Vol I, P590)

Using the spacing between lines we are able to find the boundaries of paragraphs. However, the lines of digitised pages sometimes appear inclined, indicating that the bounding boxes of the lines returned by Textract are extended beyond their actual boundaries, thus compromising the accuracy of the spacing feature. Consider for example, the left image in Fig. 5 which illustrates the proximity of bounding boxes around the final line of the paragraph and the subsequent plant name. To address this challenge, we break down each line into its individual words and retrieve their respective bounding boxes. This enables us to achieve enhanced spacing measures, ensuring greater accuracy as demonstrated in Fig. 5, right image.

**Fig. 6:**
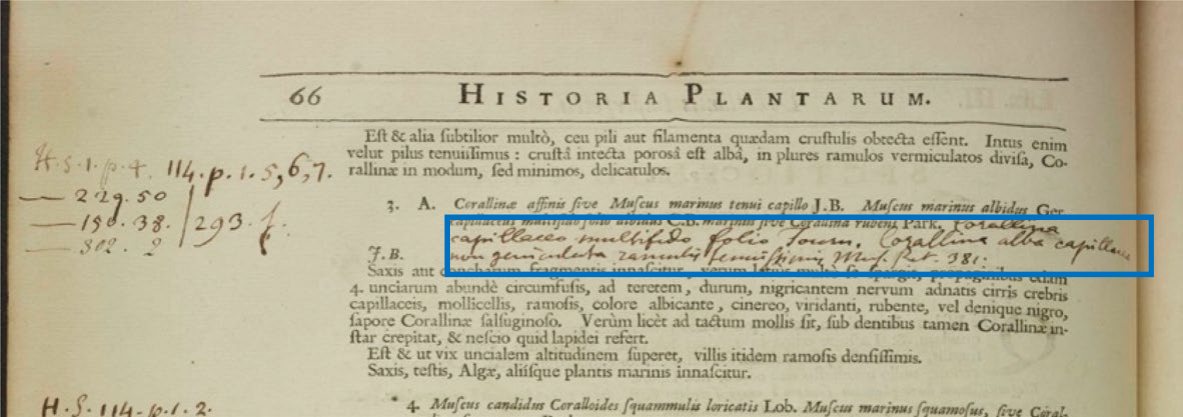
Handwritten annotations may appear between printed text (Vol I, P66)

An additional concern arises when handwritten annotations are presented within the printed text, disrupting the spacing between paragraphs, as depicted in Fig. 6. Consequently, a dedicated function was developed to overcome this issue by disregarding these handwritten annotations. This function utilises the *‘Text Types’* property returned by Textract to identify handwritten text, thus maintaining appropriate spacing positions.

**Fig. 7:**
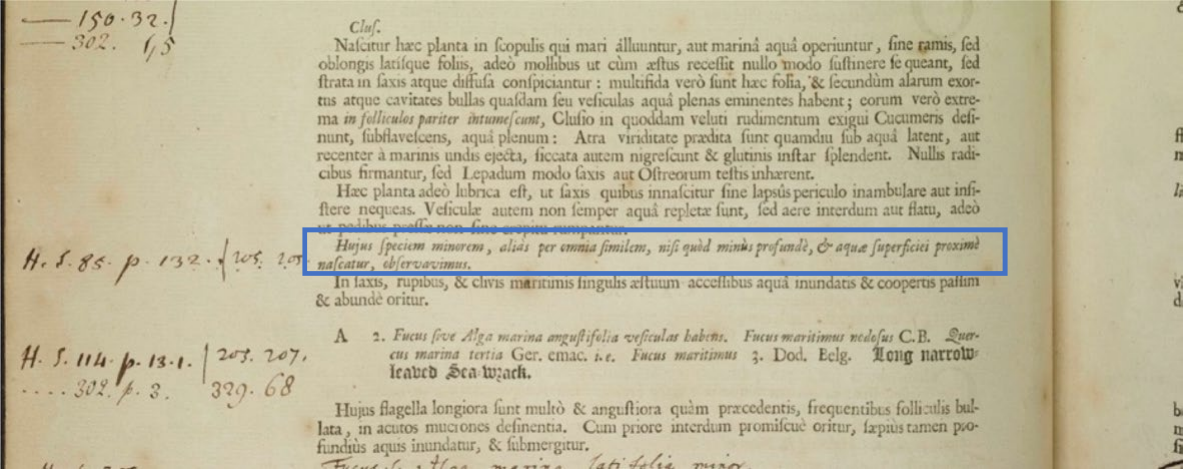
Plant name embedded inside text paragraph (Vol I, P70)

Typically, plant names are indented and spaced apart from the preceding and next paragraphs. By adopting spacing and indentation features for lines, we are able to identify plant names. When plant names are not indented, they can be confused with other paragraphs as shown in Fig. 7. In this case, to classify the paragraph as a plant name, other patterns and characteristic features are checked, for example, the number of paragraph lines or whether the first line of the paragraph starts with abbreviations or a large letter or all words are in uppercase letters.

**Fig. 8:**
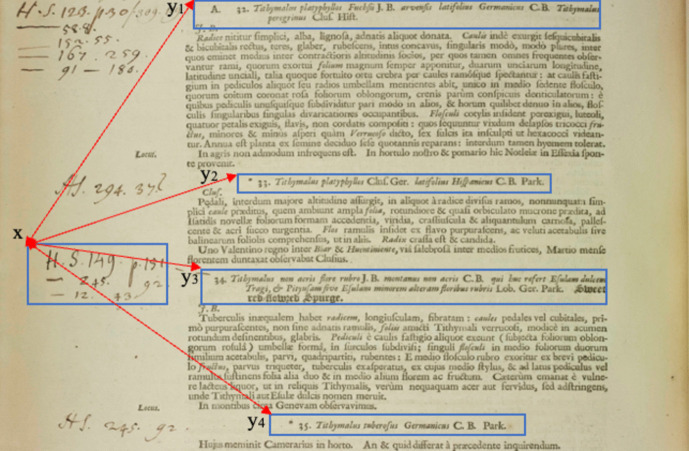
Measuring distances between specimen annotations and plant names (Vol I, P870)

Splitting the handwritten annotations on side margins into different segments is based on spacing between lines and the occurrence of initial letters (i.e. *H.S*.) The annotated specimens are linked to their corresponding plant names by calculating distances between all plant names and marginal annotations, and then the plant name is linked to the annotations with the absolute shortest distance. Calculations are based on the *Top* (y-coordinate) of the bounding box around text segments. The *Top* denotes the upper sides of the bounding box returned by Textract. Its value represents the ratio of the overall image size which is 4992 x 6668 pixels. For instance, if the *Top* coordinate of the bounding box is 1500 pixels, *Top’s* value is 0.22 (1500/6668). Consider, for example, the outlined specimens on the left margin shown in Fig. 8. By measuring the distances between the *Top* of the bounding box around these specimens and the *Tops* of all plant names boxes appearing on the page, we obtain the distances displayed below. Leading us to link these specimens to the third plant name.

$$\begin{array}{c}\left|x-y1\right|=\left|0.43-0.22\right|=0.21\\ \left|x-y2\right|=\left|0.43-0.37\right|=0.06\\ \begin{array}{c}\left|x-y3\right|=\left|0.43-0.45\right|=0.02\\ \left|x-y4\right|=\left|0.43-0.57\right|=0.14\end{array}\end{array}$$

**Fig. 9:**
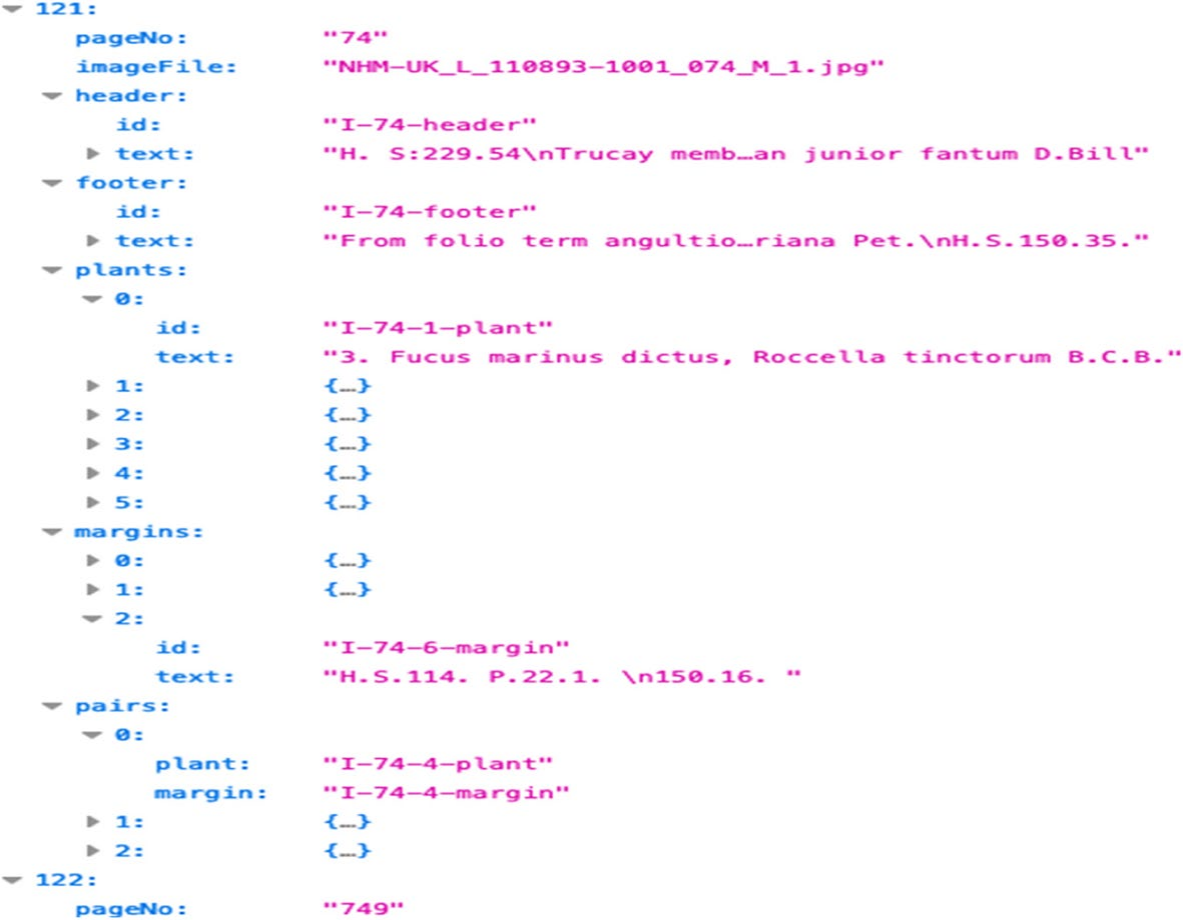
Information extracted from Vol I, P74 structured in JSON format

**Fig. 10:**
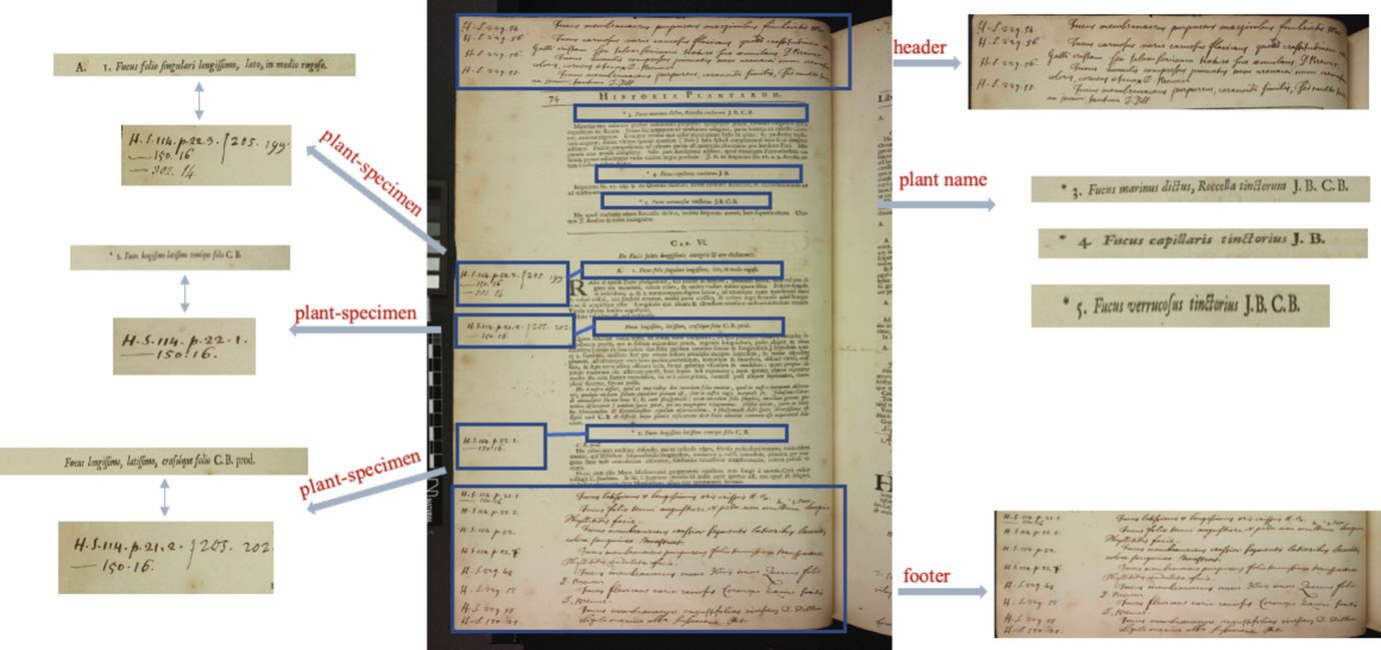
Scanned image cropped and highlighted with all relevant segments (Vol I, P74)

All captured plant names, marginalia annotations and relationships between specimens and plant names are stored in a structured file. The file follows JSON format, organised as an array of objects, where each object represents a specific page. The objects encapsulate relevant information including “header”, “footer”, “plants” and “margins” elements. Each element is assigned a unique Id and its transcribed text. Also, the object holds the “pairs” element that indicates the relationship between marginal annotations and plant names. Fig. 9 shows snippet of JSON file containing entries extracted from Volume I page 74. A corpus of cropped and marked images is also constructed, where each image has all relevant segments outlined with rectangles as illustrated in Fig. 10. Table 1 presents a summary of all elements extracted from Vol I and Vol II.

**Table 1:** Summary of the output obtained from the Data mobilisation workflow.

Element	Vol I	Vol II
Pages	983	935
Specimens	10302	7034
Headers segments	146	89
Footers segments	560	333
Margins segments	2349	1815
Printed plant names	4130	3470
Handwritten plant names	2968	1572

## Experimental results

All stages of the data mobilisation workflow were executed using Java and the Spring Cloud^1^ framework which involves invoking the Textract API^2^ and implementing the algorithm to parse Textract’s output, detect page structure, identify the relevant segments, and establish relationships. Two different experiments were conducted: the first experiment, discussed in Section 4.1, sought to measure the accuracy of text recognition generated by the Textract service. The second experiment, detailed in Section 4.2, assessed the precision in identifying the relevant segments and capturing the relationships connecting plant names to their corresponding handwritten annotations. The technical implementation of the workflow began in 2022, with the output generated by Amazon Textract being obtained in late 2022. Since Textract is a managed service by AWS and is continuously fine-tuned, repeating the text recognition experiment at a later date may yield different results. Students specialising in digital humanities at University College London contributed to creating the ground truth datasets required for these experiments (see Acknowledgements below).

### Text recognition

In this experiment, we specifically aimed to test the transcription accuracy generated by Amazon Textract, the service used to implement the Text recognition stage of the data mobilisation workflow. However, since Textract is a general-purpose service for extracting text and data from various types of documents, we anticipate that OCR & HTR systems specifically trained on historical documents may outperform it. Therefore, we also compared Textract’s performance with Transkribus which is well regarded within the digital humanities domain for its proficiency in analysing handwritten and historical documents (Nockels et al., 2022)

Transkribus features over 100 publicly available HTR models, covering different languages, script-, and font types. For the experiment with the printed text, we chose **Transkribus Print M1** (READ COOP SCE, 2022a). Created by the Transkribus Team, this covers multiple printed fonts and languages, including among others, antiqua and blackletter prints written in Latin. The model is trained on 5068310 words and the creators report a Character Error Rate (CER) of 2.20 % on the model’s validation set. For the experiment with the handwritten text segments (Headers & Footers; Side Margins) we selected **Transkribus English Handwriting M3** (READ COOP SCE, 2022b). The model is based on the handwriting of Jeremy Bentham (1748-1832), and non-specified additional English handwritten material from the 18th to 20th century. In total the training data accumulates to 2,125,253 words. The CER on the validation set is 5.10%.

A random sample of ten pages, containing around 4000 words and 22000 characters, was selected from Vol I and human reviewers transcribed the plant names and handwritten text in the footers, headers, and margins on each page. We used CER, which is calculated using Evaluate,^3^ based on Levenshtein distance. CER is a common evaluation metric used for assessing the effectiveness and reliability of OCR and HTR transcriptions CER calculates the percentage of errors made (insertion, deletions, and substitutions) in the transcribed text compared to the ground truth text (Neudecker et al., 2021). CER is calculated by dividing the total number of character errors by the total number of characters in the ground truth transcription, the resulting number is multiplied by 100. The lower the CER, the better the performance of the system. Table 2 shows CER for the printed and handwritten texts. As can be seen, the M1 and M3 Transkribus models outperformed Textract for printed names (CER of 3.1% ) and for handwritten texts in Headers & Footers (CER of 27%) respectively. Textract performed slightly better than the M3 model for handwritten numbers in the side margins (CER of 38%).

**Table 2:** Character Error Rate of Amazon Textract and Transkribus over three separate input types

	Type	Textract	Transkribus (M1)	Transkribus (M3)
Plant Names	Printed text	8.3%	3.1%	N/A
Headers & Footers	Handwritten text	40%	N/A	27%
Side Margins	Handwritten numbers	38%	N/A	39%

### Layout and relationships detection

The performance of layout and relationship detection algorithms was also investigated. Twenty pages were randomly extracted from Vol I to create the ground truth dataset. Two human experts were involved to mark (i.e. annotate) the headers, footers, and plant name segments in each page. An additional feature of the ground truth beyond annotation of the page segments was the definition of link (i.e. associative relationship) between plant names to the corresponding page margin segment holding the specimen reference to the respective Horti Siccus folio and specimen.

We used this ground truth dataset to evaluate the performance of the segmentation and relationships protocol, specifically, by establishing whether the text segments and relationships identified in the two approaches matched. We considered segments to be a match when their Intersection over Union (IoU) score exceeded 0.7. Overall, the average IoU obtained across all evaluated segments surpassed 0.82. This is like a binary classification task. The F1 is the harmonic mean of two other metrics Precision, and Recall and it is often used as a single metric of system performance. In the context of this experiment, *Precision* is the percentage of correctly recognized elements out of all recognized elements while *Recall* is the percentage of correctly recognized elements out of all actual elements in the ground truth data. The F1-Score is calculated as:

($$\text{F}1=2*\left(\text{precision}*\text{recall}\right)/\left(\text{precision}+\text{recall}\right)$$

F1-score ranges from 0 to 1, with a higher score indicating better performance. Table 3 presents the experiment results for layout and relationships detection algorithm. The highest F1 scores were found for Headers and Footers (F1=0.97); with F1 scores for side margins, plant names and relationships lower (0.86-0.91).

**Table 3:** Recall, Precision and F1-Score for Layout and relationship detection task

Element	Total Segments	Recall	Precision	F1-Score
Headers	20	0.97	0.97	0.97
Footers	20	0.95	1	0.97
Side Margins	59	0.87	0.92	0.89
Plant Names	106	0.93	0.9	0.91
Relationships	58	0.84	0.88	0.86

### Discussion

#### Text recognition

**Fig. 11:**
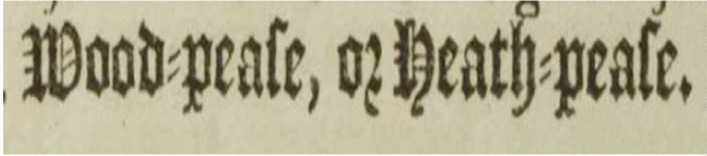
Common name written in Gothic font

**Fig. 12:**
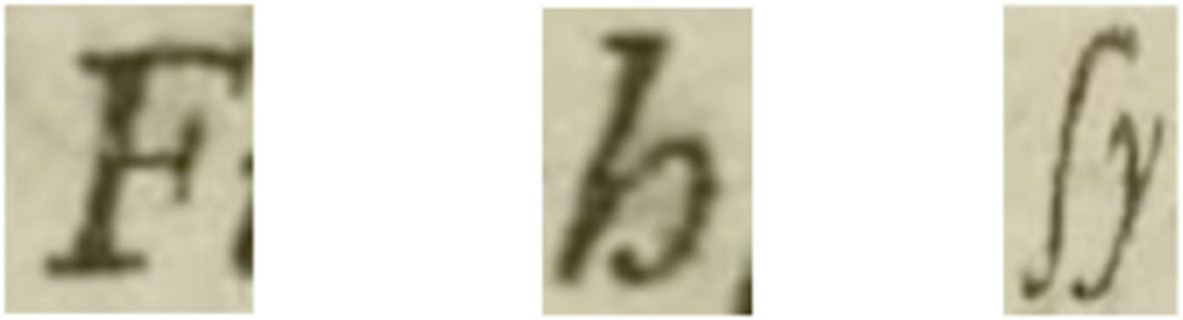
Samples of the letters that misrecognized by Textract

As outlined in Section 4.1, the evaluation of a text recognition system’s performance is based on the “distance” between the Ground Truth and the automatically recognised text. Table 2 presents the results: Textract misrecognised 8.3% of characters while Transkribus M1 model misrecognised 3.1% of characters re. Achieving a CER of 10% or below is considered highly efficient for automated transcription (Transkribus CER, n.d.). A careful examination of the misrecognized characters reveals that the primary source of errors is attributed to the usage of Gothic fonts which was used by John Ray to denote the common names in plant nomenclature as illustrated in Fig. 11. Utilising a model trained on Gothic fonts can enhance the accuracy rate for text segments typed in Gothic. However, it can also result in more errors for text written in Latin font, introducing the complexity of needing to detect the font type and switch between different models accordingly. Additionally, Textract misreads a set of printed letters, for instance, the letters shown on Fig. 12 should be transcribed as ‘F, h, fy’, yet Textract identified them as ‘J, b, sy’ respectively. This issue can be effectively resolved through a post processing stage which we will be our focus in the next phase.

**Fig. 13:**
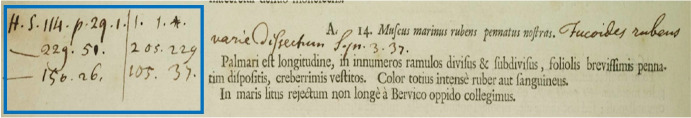
Handwritten annotations on left margin Vol I (Page 78)

**Fig. 14:**
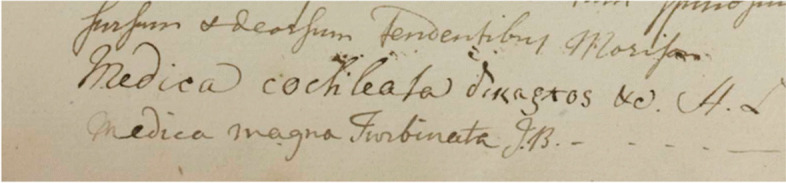
Handwritten annotations on bottom margin Vol I (Page 961)

Regarding the recognition of handwritten numbers on side margins, both Textract and M3 models exhibited poor performance with an error rate of 38% and 39% respectively. Fig. 13 demonstrates examples of handwritten specimens on the left margin of the page. The corresponding transcriptions provided by Textract and Transkribus M3 model for these references are presented in Table 4. It is crucial to identify these numbers, particularly the periods (‘.’) with 100% accuracy as they indicate specific locations in the Sloane herbarium. Currently, no HTR system can achieve the required level of accuracy. Human experts are required to accomplish this task. Fig. 14 demonstrates example of handwritten text on the bottom margin. The corresponding transcriptions provided by Textract and Transkribus M3 model are presented in Table 5.

**Table 4:** Transcriptions obtained by Textract and Transkribus M3 Model side margin annotations on Fig. 13

GT	Textract	Transkribus (M3)
H.S.114.p.29.1. 1.1.*	H. S. 114.p29.1.1. 1.A	H.S. 114. P. 291 114
-229.51. 205.229	A2g:50. 205.229	229.5. 205.229
- 150.26. 105.37	- 156.26, 105.37	150.26 10,537

**Table 5:** Transcriptions obtained by Textract and Transkribus M3 Model bottom margin annotations on Fig. 14

GT	Textract	Transkribus (M3)
Sursum & deorsum tendentibus Morison	funform + deather Tendentibay Morlison	furfun & dearfum Fendentibu Morifo
Medica cochleata δικaξλοs &c. H.L	medical cochicator dinagros &c. A.d	Medica cockleata dinacros &c. A.x
Medica magna Turbinata J.B	medica magna Inribata J.B	Medica magna Turbinata IB

#### Layout and relationship detection

The results indicate that layout detection and segment extraction algorithms performed effectively. Capturing headers and footers segments provided a high accuracy rate achieving an F1-Score of 0.97. Similarly labelling paragraph segments as plant name yielded a satisfactory performance obtaining 0.93 and 0.9 accuracy for Recall and Precision respectively. Leading to an F1-Score of 0.91. On the other hand, the side margin capture algorithm obtained a Recall of 0.87 and a Precision of 0.92 resulting in an F1-Score of 0.89. Extracting side margins segments can be rather challenging as it involves recognising handwritten texts, a process that is conducted by Textract. If the quality of the image is poor and the margins are faded, it becomes difficult for Textract to recognize the characters accurately leading to misidentification of the margin segment.

**Fig. 15:**
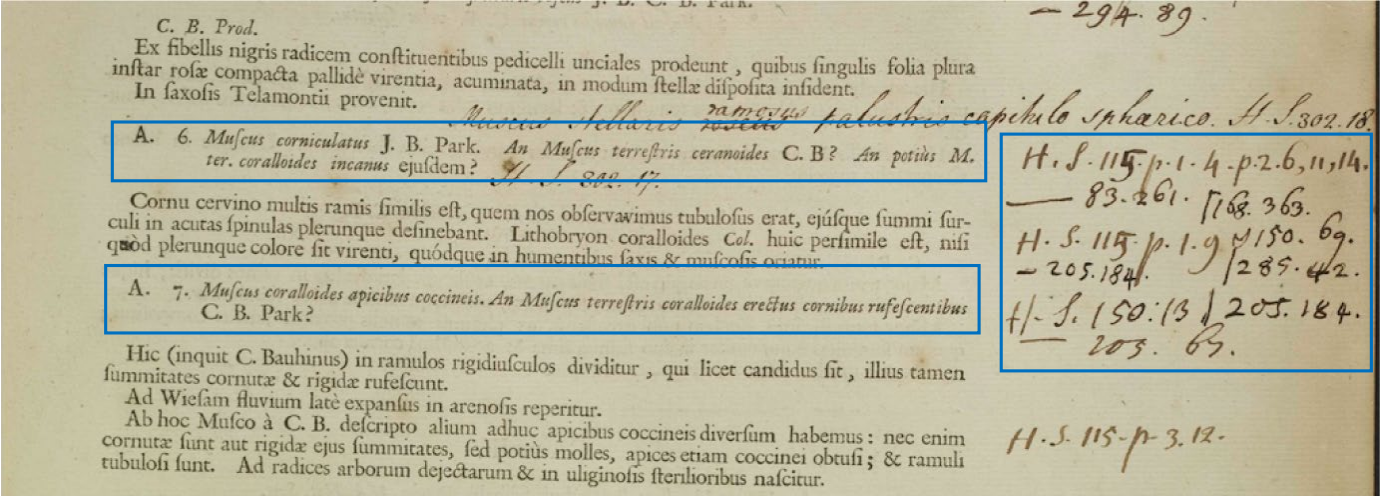
Challenges in establishing clear connections between specimen annotations on right margin and plant names

On the other hand, predicting the relationships that link specimens on side margins to their correspondence plant names depends heavily on the accuracy of identifying the side margins and plant name segments. In some cases, the handwritten specimens intersect with each other and even humans can struggle to linking the specimen annotations to the correct plant name. An example of marginalia that exhibits this issue can be observed in Fig. 15 where it is challenging to determine which specimens of the handwritten text on the right margin refer to plant A.6 and which refer to plant A.7. The relations detection algorithm obtained a Recall of 0.84 and Precision of 0.88. Overall, the algorithm is highly accurate when the image is clear and plant name segments are identified correctly.

## Conclusion and future work

The aim of this study is to establish a workflow to extract data from Hans Sloane’s copy of John Ray’s *Historia Plantarum* and thus provide, for the first time, a resource for searching plant specimens in the Sloane Herbarium by the species name used by Ray. *Historia Plantarum* remains the primary taxonomic index to the Sloane Herbarium and there is currently no digitally searchable representation of the data it contains. The mobilisation of these data therefore has the potential to bring about a step-change in access to these data.

To achieve the aims of this study, it is essential to capture various types of information: (i) the plant names listed in the main printed content of text, (ii) the handwritten references located in heads, footers, and side margins, and (iii) the connections between plant names and their related specimen annotations. Given the complexity of this task and the NHM’s requirement for 100% accuracy in recognising handwritten numbers, current OCR/HTR systems alone are insufficient, requiring a tailored solution.

In this study, a data mobilisation workflow was proposed to leverage the output of Textract. The proposed workflow performed well, achieving high accuracy of layout detection and relationships detection, particularly for header and footer segments. However, the performance of text recognition is not satisfactory, and we plan to improve the accuracy of printed and handwritten text recognition obtained through the OCR and HTR by passing output through a post processing stage that could involve integrating advanced techniques such as Large Language Models (LLMs) like GPT-based models or transformer-based models like Transkribus Text Titan. Additionally, in May 2023 the Sloane Lab HTR model was developed in Transkribus (desktop client) based on the images and transcriptions of Sloane's Catalogue of Miscellanies (folio 3-152, recto and verso). It is expected that the incorporation of this model would yield improved results since it is based on a training set that was predominantly written by Sloane.^4^

We also plan to involve experts to go around the handwritten characters The integration of human expertise and machine technology plays a crucial role for the successful digitisation of historical documents, particularly those with complex formats. One example is the Old Weather project (Blaser, 2016) , hosted on Zooniverse, which aimed to digitise historical ship logbooks to study climate change research. While machine learning models are used to pre-process the data, human volunteers help interpret hard-to-read handwritten entries, filling in gaps left by the machines. Another notable project is Transcribe Bentham** (**Causer et al., 2018), an initiative launched by UCL, which used OCR to digitise the manuscripts of Jeremy Bentham but relies on volunteers to correct the errors (the staff of the project going on to play an important role in Transkribus). This collaborative framework would benefit our project in the scenario that the output of OCR/HTR systems provide a draft that human annotators can quickly correct, rather than transcribing from scratch. This reduces the manual effort required where reviewers can focus on correcting specific errors, such as misrecognized characters, or ambiguous handwriting. Additionally, the confidence score returned by Textract for each word can flag areas of uncertainty or ambiguity, guiding us to specific sections that need attention. Another advantage is the user interface in man-machine collaborations which makes human involvement more enjoyable by designing customised annotation workflows and tools that present progress and provide immediate feedback.

## Data Availability

Datasets generated during the current study are not yet publicly available.

## Footnotes

1 Spring Cloud Dependencies V 2021.0.5.

2 AWS Java SDK:Textract V 2.18.20.

3 Evaluate Library V 0.4.1, Hugging Face.

4 https://github.com/sloanelab-org/HTR-Model
